# Alteration of fatty acid oxidation by increased CPT1A on replicative senescence of placenta-derived mesenchymal stem cells

**DOI:** 10.1186/s13287-019-1471-y

**Published:** 2020-01-03

**Authors:** Jin Seok, Hyun Sook Jung, Sohae Park, Jung Ok Lee, Chong Jai Kim, Gi Jin Kim

**Affiliations:** 1 Department of Biomedical Science, CHA University, 689, Sampyeong-dong, Bundang-gu, Seongnam-si, Gyeonggi-do Republic of Korea; 2 Department of Anatomy, Korea University College of Medicine, Seoul, Republic of Korea; 3 Department of Pathology, University of Ulsan College of Medicine, Asan Medical Center, Seoul, Republic of Korea

**Keywords:** Placenta-derived mesenchymal stem cell, Senescence, Mitochondria, Fatty acid, CPT1A

## Abstract

**Background:**

Human placenta-derived mesenchymal stem cells (PD-MSCs) are powerful sources for cell therapy in regenerative medicine. However, a limited lifespan by senescence through mechanisms that are well unknown is the greatest obstacle. In the present study, we first demonstrated the characterization of replicative senescent PD-MSCs and their possible mitochondrial functional alterations.

**Methods:**

Human PD-MSCs were cultured to senescent cells for a long period of time. The cells of before passage number 8 were early cells and after passage number 14 were late cells. Also, immortalized cells of PD-MSCs (overexpressed hTERT gene into PD-MSCs) after passage number 14 were positive control of non-senescent cells. The characterization and mitochondria analysis of PD-MSCs were explored with long-term cultivation.

**Results:**

Long-term cultivation of PD-MSCs exhibited increases of senescent markers such as SA-β-gal and p21 including apoptotic factor, and decreases of proliferation, differentiation potential, and survival factor. Mitochondrial dysfunction was also observed in membrane potential and metabolic flexibility with enlarged mitochondrial mass. Interestingly, we founded that fatty acid oxidation (FAO) is an important metabolism in PD-MSCs, and carnitine palmitoyltransferase1A (CPT1A) overexpressed in senescent PD-MSCs. The inhibition of CPT1A induced a change of energy metabolism and reversed senescence of PD-MSCs.

**Conclusions:**

These findings suggest that alteration of FAO by increased CPT1A plays an important role in mitochondrial dysfunction and senescence of PD-MSCs during long-term cultivation.

## Background

Human mesenchymal stem cells (hMSCs) are adult multipotent stem cells that can be isolated from various tissues, including the bone marrow, adipose tissue, muscle, and placenta, which are currently considered as a powerful source for stem cell transplantation in the field of regenerative medicine [1]. Human placenta-derived mesenchymal stem cells (PD-MSCs) obtained from fetal tissue origin have emerged as a new alternative source of MSCs, which have advantages for multipotent differentiation, strong immunosuppressive properties, and easily accessible to obtain abundant cells in vitro, moreover free from ethical concerns [2]. However, cultured primary human cells, including hMSCs, undergo a limited number of cell division and reach a state of irreversible growth arrest in a process called cellular senescence [3]. Senescence of MSCs is considered to be the major disadvantage of cell-based therapy. The major characteristics or molecular changes of senescent MSCs are the telomere shortening or dysfunction, DNA damage foci, enlarged cell size including flattened appearance, increased senescence-associated β-galactosidase (SA-β-Gal) activity, oxidative stress, deranged mitochondrial metabolism, as well as altered signaling of sirtuins, insulin/insulin-like growth factor-1 (IGF-1) [4, 5]. In addition to these markers, increased autophagic vacuole with enhanced β-Gal activity is associated with senescent fibroblast [6]. A previous study demonstrated autophagy is activated during long-term MSC culture and autophagic activity is a requirement for maintaining the senescent state of MSCs [7]. Moreover, the biological phenomena of autophagy are similar to those of senescence including DNA damage, telomere shortening, and induction of p53 and p21 involving the generation of reactive oxygen species (ROS) in chemotherapy-induced senescence [8, 9]*.*

Mitochondria are double membrane-bound dynamic organelles and generate ATP through oxidative phosphorylation as an important source of cellular energy. Mitochondrial ATP synthesis is coupled with respiration, which also produces a certain amount of reactive oxygen species (ROS), including hydrogen peroxide, superoxide, hydroxyl radicals, and singlet oxygen, as byproducts of the normal cellular metabolism [10]. Excess of ROS can cause DNA damage, lipid peroxidation, and oxidative modification of proteins, which are detrimental to cell function [11]. Thus, proper mitochondrial function is an important role in maintaining homeostasis and engagement of appropriate stress responses both at the level of the cell and the entire organism. Recently, a number of studies associated with mitochondrial metabolism or dysfunction-related stem cell biology have risen, especially in aging or self-renewal ability of stem cells [10, 12, 13]. Interestingly, enlarged cell morphology reflecting increased cell mass, one of the characteristics of senescent cells, was accompanied by increased membrane lipid content and lipid biosynthesis in both stress-induced senescence and replicative senescence [14].

Carnitine palmitoyl transferase 1 (CPT1) is a transmembrane enzyme of the mitochondrial outer membrane, which converts long-chain fatty acyl-CoA to long-chain acylcarnitine, following which carnitine acylcarnitine translocase transports the fatty acid across the inner mitochondrial membrane and then enter fatty acid β-oxidation. CPT1 is inhibited by malonyl-CoA, an intermediate of lipogenesis, synthesized by acetyl-CoA carboxylase (ACC) [15]. There are three tissue-specific isoforms of the CPT1 family, CPT1A (liver form), CPT1B (muscle form), and CPT1C (brain form) [16]. Quijano et al. demonstrated that oncogene-induced senescence (OIS) cells exhibited a marked elevation in fatty acid oxidation (FAO), and knockdown of CPT1A expression reduced oxygen consumption and basal metabolic rate of OIS cells [17]. The human placenta utilizes fatty acids as a significant metabolic fuel and derives energy from FAO [17].

Currently, researches on mitochondrial dysfunction and cellular senescence have been reported in many aspects, but there have been no studies on senescent PD-MSCs and their associated mitochondrial dysfunction. In this study, we analyzed the alterations in overall cellular metabolism including autophagy and mitochondrial function during replicative senescence of PD-MSCs, and specifically focused on the effect of FAO by CPT1A inhibition in the senescence process of PD-MSCs during long-term cultivation.

## Methods

### Mesenchymal stem cell isolation and culture

Placenta-derived mesenchymal stem cells (PD-MSCs) were isolated from the chorionic plate of placentas as described previously [18] and cultured in alpha-minimum essential medium (α-MEM; Hyclone, South Logan, Utah) supplemented with 1% penicillin/streptomycin (GIBCO-BRL, Langley, Oklahoma), 25 ng/mL FGF-4 (Peprotech, Rocky Hill, NJ, USA), 1 μg/mL heparin (Sigma-Aldrich, St. Louis, MO, USA), and 10% fetal bovine serum (FBS; GIBCO-BRL) at 37 °C in a humid atmosphere containing 5% CO_2_. The cells before passage number 8 were “early,” and after passage number 14 were “late.” Immortalized cells of PD-MSCs after passage number 14 were obtained with human telomerase reverse transcriptase (hTERT) overexpression as a positive control of non-senescent cells.

### Cell treatment with etomoxir and siRNA

The target gene was inhibited with etomoxir (Sigma-Aldrich) and CPT1A siRNA (5′-GACGUUAGAUGAAACUGAAUU-3′, 5′-UUCAGUUUCAUCUAACGUCUU-3′) (Invitrogen Corporation, San Diego, CA, USA). In order to know the conditions, cells were seeded in a six-well plate at a density 5 × 10^4^ cells/well. After cultured with a plate for 24 h, the cells were treated with 200 μM etomoxir and 10 nM CPT1A siRNA for 24 h.

### Differentiation into mesodermal lineages

For in vitro differentiation into adipoblast, PD-MSCs were plated at a density of 2.5 × 10^4^ cells/30 mm dish and cultured in adipogenic induction medium containing 1 μM dexamethasone, 0.5 mM isobutyl methylxanthine (IBMX), 0.2 mM indomethacin, 1.7 μM insulin (Sigma-Aldrich), 10% FBS (GIBCO-BRL), and 1% penicillin/streptomycin (GIBCO-BRL) with medium changes three times a week. After 21 days, PD-MSCs were fixed with 4% paraformaldehyde (PFA) and were analyzed by Oil-Red O (Sigma-Aldrich) staining to induce osteogenic differentiation, and PD-MSCs were plated at a density of 2.5 × 10^4^ cells/30 mm dish and cultured in osteogenic induction medium containing 1 μM dexamethasone, 10 mM glycerol-2-phosphate (Sigma-Aldrich), 50 μM L-ascorbic acid 2-phosphate (Sigma-Aldrich) 10% FBS, and 1% penicillin/ streptomycin with medium changes three times a week. After 21 days, calcium deposits in PD-MSCs were evaluated by von Kossa staining using 5% silver nitrate (Sigma-Aldrich) under light for 1 h. The differentiated cells for osteogenic and adiogenic were marked by arrowheads.

### Cell proliferation assay

The cell proliferation was measured using Ez-Cytox (WST-1 assay) cell viability assay kit (Daeil Lab Service, Seoul, South Korea). Each PD-MSCs was seeded into 96-well plate (2 × 10^3^ cells/well) and cultured for 1, 2, 3, and 4 days. Then 100 μl of EZ-cytox solution was added to each well and incubated at 37 °C for 2 h. After incubation, the conditioned medium was transferred to 96-well plates and the absorbance was measured by an Epoch microplate spectrophotometer (Biotek, VT, USA) at 450 nm.

### Total RNA extraction and real-time PCR analysis

Total RNA was extracted from 80% confluent cells using TRIzol reagent (Ambion, CA, USA) following the manufacturer’s protocol. One microgram of total RNA was used for cDNA synthesis and first-strand cDNA was produced using Oligo dT and Superscripts III Reverse Transcriptase (Invitrogen Corporation) according to the manufacturer’s instructions. To analyze the markers related to stemness, the cDNA was quantified using SYBR green (Roche Diagnostics, Indianapolis, IN, USA) in a PCR machine (Refurbished Biometra Thermal Cyclers; LabRepCo, Horsham, Pennsylvania). Primers were targeted against Oct4 (5-CCTCACTTCACTGCACTGTA-3, 5-CAGGTTTTCTTTCCCTAGCT-3), Nanog (5- TTCTTGACTGGGACCTTGTC-3, 5-GCTTGCCTTGCTTTGAAGCA-3), Sox2 (5-CCCAGCAGACTTCACATGT-3, 5-CCTCCCATTTCCCTCGTTTT-3), hTERT (5-GAGCTGACGTGGAAGATGAG-3, 5-CTTCAAGTGCTG TCTGATTCCAATG-3), and hGAPDH (5-CTCCTCTTCGGCAGCACA-3, 5-AACGCTTCACCTAATTTGCGT-3). Target sequences were amplified by using the following conditions: 95 °C for 10 min, followed by 40 cycles of 95 °C for 10s, at 60 °C for 20s. All reactions were performed in triplicate. hGAPDH was used as an internal control gene for calculation of a qRT-PCR normalization factor. Data were analyzed by the comparative CT method. To analyze the markers related to differentiation, the cDNA was amplified using Taq DNA polymerase (Solgent, Daejeon, South Korea). Primers were targeted against osteocalcin (5-GCAGCGAGGTAGTGAAGAGA-3, 5-CGATGTGGTCAGCCAACT-3), Adipsin (5-GCTGGAGTTCAGTGGTGTGA-3, 5-ACCAACCTGACGAATGTGGT-3), and GAPDH (5-TTATTATAGGGTCTGGGATG-3,5-ACACTGAGGACCAGGTTGTC-3). Target sequences were amplified by using the following cycling conditions: 95 °C for 15 min, followed by 40 cycles of95 °C for 20 s, 58 °C and 59 °C for 40 s, 72 °C for 1 min, and a final extension at 72 °C for 5 min. PCR products were mixed with loading dye (Cha Biomed, Seongnam, South Korea) and analyzed by electrophoresis on a 1.2% agarose gels (Lonza, Basel, Switzerland). The agarose gels were visualized with ChemiDoc (Bio-Rad Laboratories, Hercules, CA, USA).

### Protein extraction and Western blotting

Cells were lysed in a lysis buffer (50 mM Tris pH 7.4, 150 mM NaCl, 1% Triton X-100, and 0.1% SDS) containing protease inhibitor cocktail (Roche, IN, USA) and phosphatase inhibitor cocktail II (A.G scientific, San Diego, USA). The protein concentration in each lysate was measured using a BCA protein assay kit (Pierce, Massachusetts, USA). Equal amounts of protein (20–50 μg) were separated using 6–15% sodium dodecyl sulfate-polyacrylamide gels electrophoresis (SDS-PAGE) and transferred onto polyvinylidene difluoride membranes (PVDF; Bio-Rad Laboratories) using a trans-blot system (Bio-Rad Laboratories). The membranes were blocked with 3% skim milk and incubated overnight at 4 °C with the following primary antibodies specific for p21, PPARα (Abcam, USA), p53, ATG5-12, Bax, Bcl2 (Santa Cruz Biotechnology, USA), p-p44/42 MAPK (Thr^202^/Tyr^204^), p-AMPK (Thr^172^), AMPK, p-ACC (Ser^79^), ACC, p-Akt (Ser^473^), Akt, CPT1A, PI3 Kinase p110α, PI3 Kinase p85 (Cell Signaling Technology, USA), LC3I, II (Novus Biologicals, USA), GAPDH (Ab Frontier, South Korea), and α-tubulin (Oncogene). Subsequently, the membranes were washed several times with TBS-T and incubated with horseradish peroxidase (HRP)-conjugated secondary antibodies (anti-mouse and anti-rabbit IgG; Bio-Rad Laboratories) and developed with the enhanced chemiluminescence (ECL) detection reagents (Amersham plc, cataway, NJ). The intensity readings for Western blot band were measured using an Image J program (NIH, Bethesda, Maryland). The fold change value of the intensity is a comparative value of gene expression in each late and hTERT+ groups based on the gene expression value in the early group as 1.

### Cell cycle analysis

For cell cycle analysis, PD-MSCs were fixed in 70% ice-cold ethanol at 4 °C overnight. Cells were then centrifuged, washed and re-suspended in 1 ml cold phosphate-buffered saline (PBS) containing 1% BSA and RNase (50 μg/ml). Subsequently, cells were stained with propidium iodide (PI; 5 μg/ml; Sigma-Aldrich) for 15 min at 37 °C in the dark. The intensity of fluorescence was analyzed by a BD FACS Vantage SE Cell Sorter (BD Bioscience Pharmingen, San Diego, CA, USA). The percentage of cells in the G1, S, and G2/M were analyzed using Cell Quest software (BD Biosciences).

### Senescence associated β-galactosidase (SA-β gal) assay

The senescence-associated β-galactosidase activity was detected using the SA-β-gal staining kit (Cell signaling, Danvers, USA) according to the manufacturer’s instructions. Briefly, cells were washed with PBS and fixed for 10–15 min in 1X fixative solution at room temperature. After washing with PBS, the cells were incubated overnight at 37 °C with 1X SA-β-gal staining solution (pH 6.0). The percentage of positive cells and cell size were analyzed with a microscope via a high and digital camera (Nikon instrument, Nikon Inc., Melville, NY, USA) and image J program (NIH). Positive signals of SA-b-gal staining were marked by an arrowhead.

### Bioenergetic analysis using the XF24 analyzer

To determine the extracellular acidification rate (ECAR) and oxygen consumption rate (OCR) of senescent PD-MSCs, a XF24 analyzer (Seahorse Bioscience, Billerica, MA, USA) was used according to the manufacturer’s protocol. PD-MSCs (5 × 10^3^ cells/well) were seeded in the XF24 cell culture plates and incubated at 37 °C with 5% CO2 for 24 h. The four metabolic inhibitors for analyses were oligomycin, 2-deoxyglucose (2-DG), carbonyl cyanide p-(trifluoromethoxy) phenylhyrazone (FCCP), and rotenone. ECAR, an indicator for glycolysis, was measured under basal conditions followed by the addition of 10 mM glucose, 1.0 μM oligomycin, and 50 mM 2-DG. OCR was detected under basal conditions followed by the sequential addition of oligomycin (1.0 μM), FCCP (0.5 μM), and rotenone (0.5 μM) as an indicator of mitochondrial respiration.

### MitoSox Red and MitoTracker Green staining

PD-MSCs were stained with MitoSox Red and MitoTracker Green (Invitrogen Corporation) to quantify mitochondrial superoxide production and mitochondrial content, respectively. PD-MSCs (1.3 × 10^4^ cells/well) were seeded into 24-well culture plates and washed with Hanks’ balanced salt solution (HBSS). The plates were incubated with 3uM MitoSox Red (Invitrogen Corporation) and 100 nM MitoTracker Green (Invitrogen Corporation) for 40 min at 37 °C. Cells were then washed with HBSS and incubated with 1 μg/ml diamidino-phenylindole hydrochloride (DAPI; Sigma Aldrich) for 1 min at RT. Fluorescence images were obtained using a confocal microscope (Leica TCS SP5 microscope; Leica microsystems, Wentzler, German, × 100 magnifications). Positive signals of targeted fluorescence were marked by an arrowhead in our data.

### Reactive oxygen species (ROS) measurement

The ROS levels in cells were measured using 2′,7′-dichlorofluorescein diacetate (DCF-DA). PD-MSCs (5 × 10^3^ cells/well) were seeded in the 96-well cultured for 24 h and treated with 50uM DCF-DA for 30 min. After the cells were washed with HBSS twice, the fluorescence intensity at 535 nm was measured with excitation at 485 nm using an Infinite® 200 Microplate Reader (Tecan m200; Tecan trading, Männedorf, Switzerland).

### Mitochondrial membrane potential

Mitochondrial membrane potential was determined by 5,5′,6,6′-tetrachloro-1,1′,3,3′-tetraethylbenzimidazolylcarbocyanine iodide (JC-1; Invitrogen Corporation) according to the manufacturer’s instructions. PD-MSCs (5 × 10^3^ cells/well) were seeded into 96-well culture plate for 24 h and incubated with 2 μg/ml JC-1. The fluorescence intensity was measured at 485/530 nm for JC-1 monomer and at 535/590 nm for J-aggregates, using in an Infinite® 200 Microplate Reader (Tecan m200). Results are shown as a ratio of red (535/590 nm) to green (485/530 nm) fluorescence from JC-1.

### Mitochondrial mass determination

The mitochondrial mass was evaluated using 10-*N*-nonyl-acridine orange reagent (NAO; Invitrogen Corporation) according to the manufacturer’s instructions. PD-MSCs (5 × 10^3^cells/well) were seeded into 96-well culture plates for 24 h and incubated with 10 μM NAO for 30 min. After the cells were washed twice with PBS, the fluorescence intensity was measured at 485 nm/530 nm using in an Infinite® 200 Microplate Reader (Tecan m200).

### ATP production assay

The ATP levels were measured using an ATP assay Kit (Abcam, Cambridge, MA, USA) according to the manufacturer’s instructions. Briefly, PD-MSCs (5 × 10^5^ cells/well) were seeded into 100 mm dishes for 24 h. The cells were washed with cold 1xPBS and suspended in 100 μl of ATP assay buffer in ice. After centrifugation, the supernatant was collected. ATP concentration was at 570 nm using an Epoch microplate spectrophotometer (Biotek).

### Statistical analysis

All experiments were performed at least three times. Data are expressed as means ± standard error of mean (± SEM). Statistical significance between early passage versus late passage and control group with or without etomoxir and CPT1A siRNA was determinate using the Student’s *t* test and Sigma plot (Systat Software Inc.). For all analyses, *P* < 0.05 was considered statistically significant.

## Results

### Characterization of senescent PD-MSCs during long-term cultivation

**Fig. 1:**
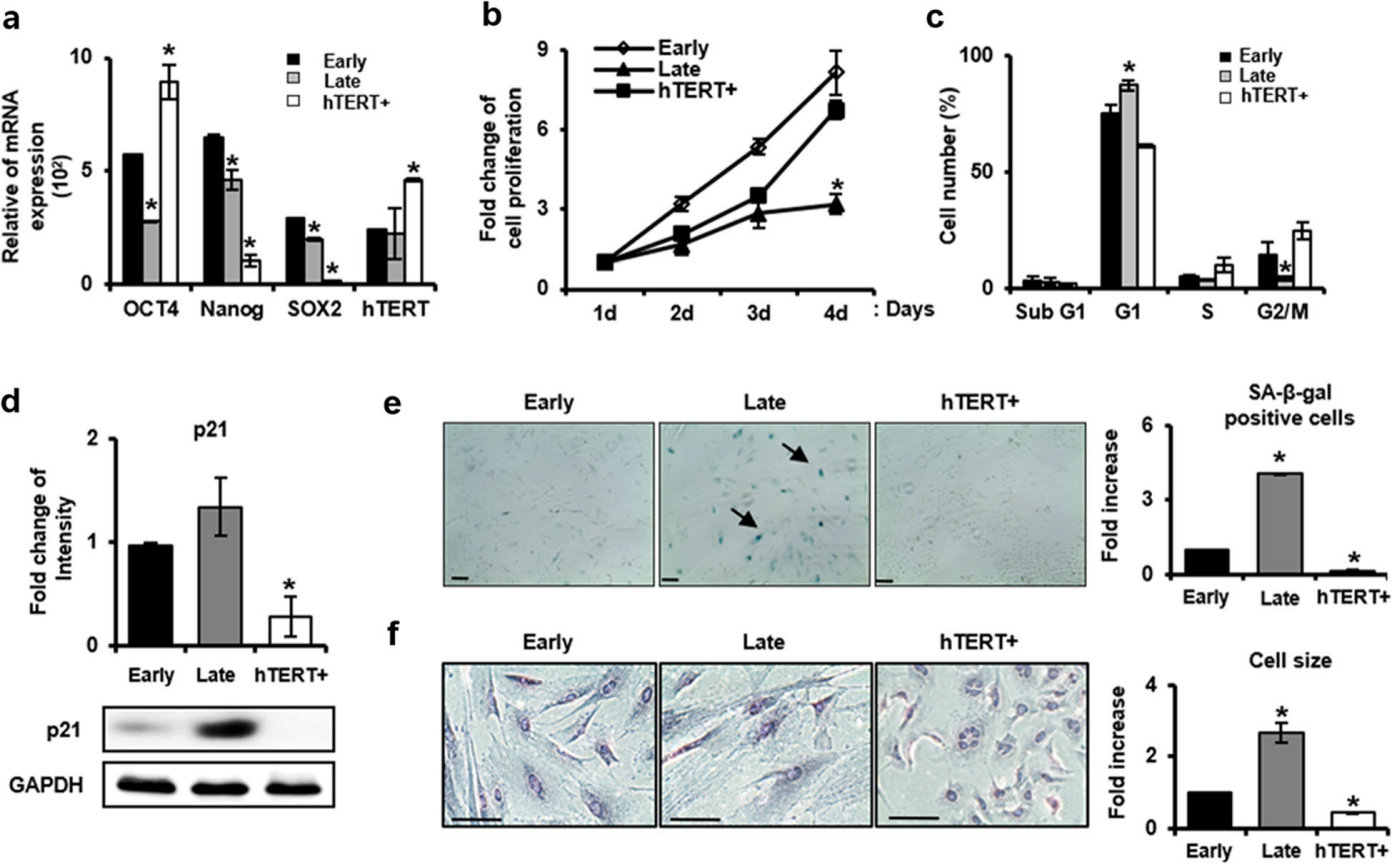
Characterization of senescent PD-MSCs during long-term cultivation. **a** Expression of stemness related genes was analyzed by qRT-PCR in early and late passage PD-MSCs. **b** In vitro proliferation of early and late passage PD-MSCs for 4 days were analyzed by Ez-Cytox cell viability assay kit. **c** Quantitation of cells in the respective phases of cell cycle by FACS analysis. **d** Expressions of p21 protein were assayed by Western blotting in early and late passage PD-MSCs. **e** Senescence of PD-MSCs was detected by senescence-associated-β-galactosidase staining (magnification, × 20). **f** The enlarged cell size was significantly counted and observed in late passage PD-MSCs by Image J program (magnification, × 200). The data were representative of three independent experiments and expressed as means ± S.D. An asterisk indicates *P* < 0.05 versus early passage

**Fig. 2:**
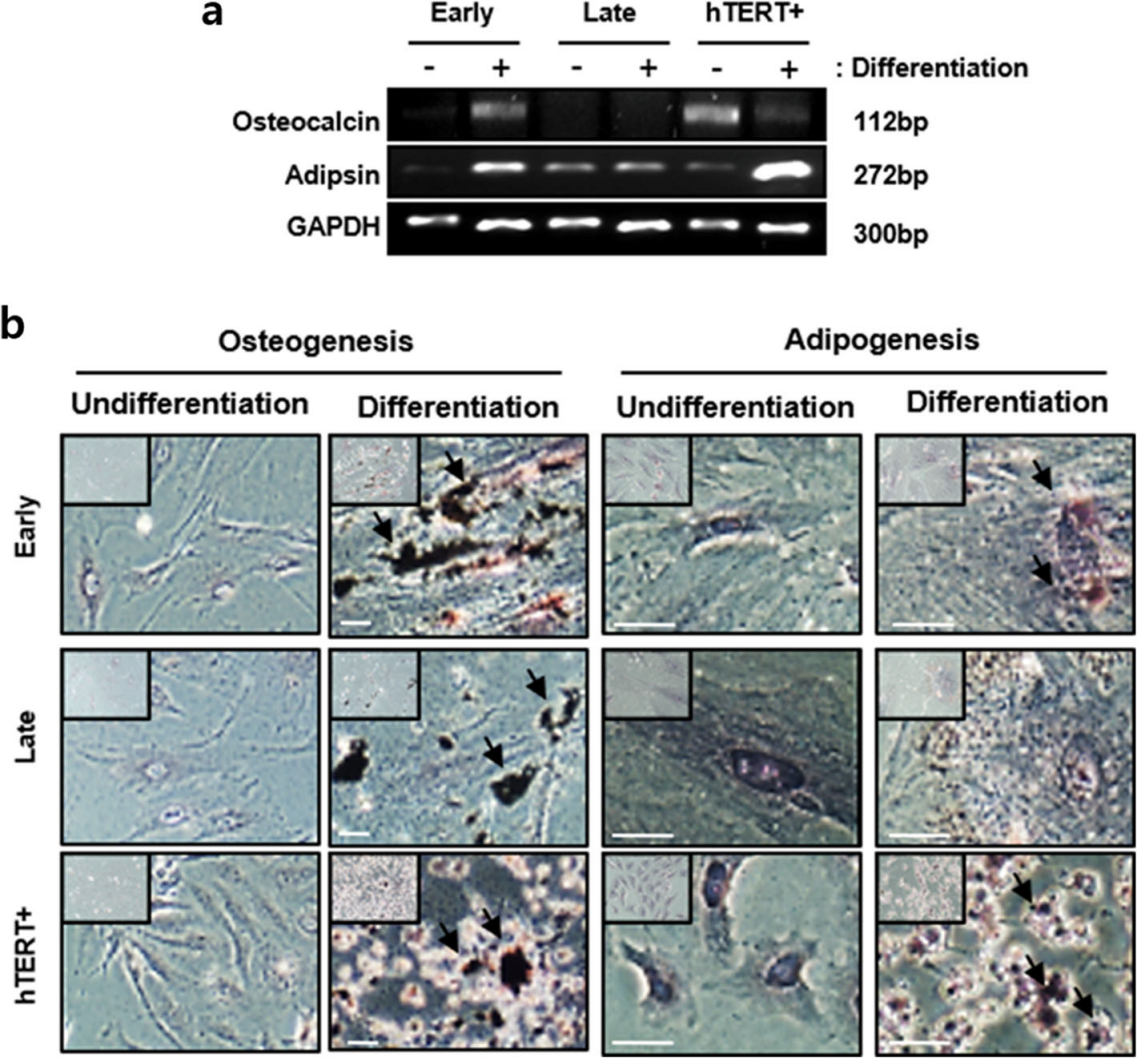
Differentiation potential of senescent PD-MSCs. **a** After early and late passage PD-MSCs and hTERT+ were differentiated for 2 weeks, the expression of genes related to osteogenesis and adipogenesis was measured by RT-PCR. **b** Osteogenic and adipogenic lineages were determined by von Kossa (magnification, × 100) and Oil-Red O staining (magnification, × 200), respectively. The data were representative of three independent experiments

To analyze the characteristics of in vitro senescent PD-MSCs during long-term cultivation, we compared differences in various biological markers related to senescence among early and late passage of PD-MSCs, and hTERT+ PD-MSCs as a positive control of non-senescent or immortalized cells by human TERT gene modification in PD-MSCs. First, stem cell markers in early and late passage of PD-MSCs were identified by qRT-PCR analysis. As shown in Fig. 1a, the expression levels of stemness genes, OCT4, Nanog, SOX2, and hTERT showed a statistically significant decrease at late passage compared to early passage. Although Nanog and SOX2 mRNA expression was reduced, hTERT+ PD-MSCs also showed a significant increase in Oct-4 and hTERT, suggesting that immortalized PD-MSCs still retain stemness potential. In addition, a decrease in proliferation rate for 4 days was observed in PD-MSCs at late passage compared to early passage and hTERT+ cells (Fig. 1b). The cell cycle distribution of PD-MSCs was determined by FACS analysis. At late passage, the percentage of G1 cells was significantly increased up to 87.2%, indicating G1 arrest, compared to early passage (75.3%), even though no difference was observed in the percentage of sub-G1 cells in both PD-MSCs. Concomitant with this, the percentages of cells in S and G2/M populations were reduced from 5.1% (early) to 3.3% (late) and 14.5% (early) to 4.3% (late), respectively. However, the control hTERT+ cells showed no G1 arrest accompanied with the increase of S phase and G2/M population (Fig. 1c). We then investigated the expression level of p21, known as an inhibitor of important molecules for G1/S transition as well as typical senescence marker, at early and late passages in PD-MSCs. As expected, late PD-MSCs showed upregulation of p21 compared to early PD-MSCs, whereas p21 protein was hardly detected in hTERT+ (Fig. 1d). In contrast, the p53 expression level, known as a tumor suppressor gene and a regulator of p21, was dramatically decreased in late PD-MSCs, whereas markedly increase in hTERT PD-MSCs (Additional file 1: Figure S1). The senescent status of late PD-MSCs was confirmed by the senescence-associated β-galactosidase (SA-β-gal) staining assay showing an increased activity of the lysosomal β*-*galactosidase specifically in senescent cells. We also examined morphological changes of senescent PD-MSCs (Fig. 1e). In late passage PD-MSCs, the number of blue positive β-gal staining was approximately 2.67-fold higher than in the early and cell size enlarged 4.05-fold and flattened (Fig. 1f). However, the expression of SA-β-gal was hardly observed in hTERT+ cells and there was no change in cell morphology. To examine whether long-term in vitro culture affects the multilineage differentiation potential, the cells were induced to differentiate into osteocytes and adipocytes. As shown in Fig. 2a, the mRNA expressions of osteocalcin and adipsin, which are osteocyte and adipocyte markers, respectively, were enhanced significantly in early PD-MSCs after differentiation induction. However, the expression of osteocalcin was not observed in late PD-MSCs before and after induction, and adipsin mRNA expression was maintained at a constant level. Similarly, osteogenic and adipogenic differentiation potential assessed by von Kossa and Oil Red-O staining, respectively, were reduced in late PD-MSCs than early. Interestingly, the mRNA expression of osteocalcin was increased in undifferentiated condition compared to differentiated condition and the mRNA expression of adipsin was highly increased in differentiated condition compared to undifferentiated condition in hTERT+ with higher self-renewal potential (Fig. 2b). Taken together, PD-MSCs during prolonged in vitro culture undergo senescence exhibiting reduced proliferation rate by G1 arrest and lowered differentiation potential, and the overexpression of hTERT helps PD-MSCs escaping from replicative senescence.

### Effect of cell survival and death pathway in senescent PD-MSCs during long-term cultivation

**Fig. 3:**
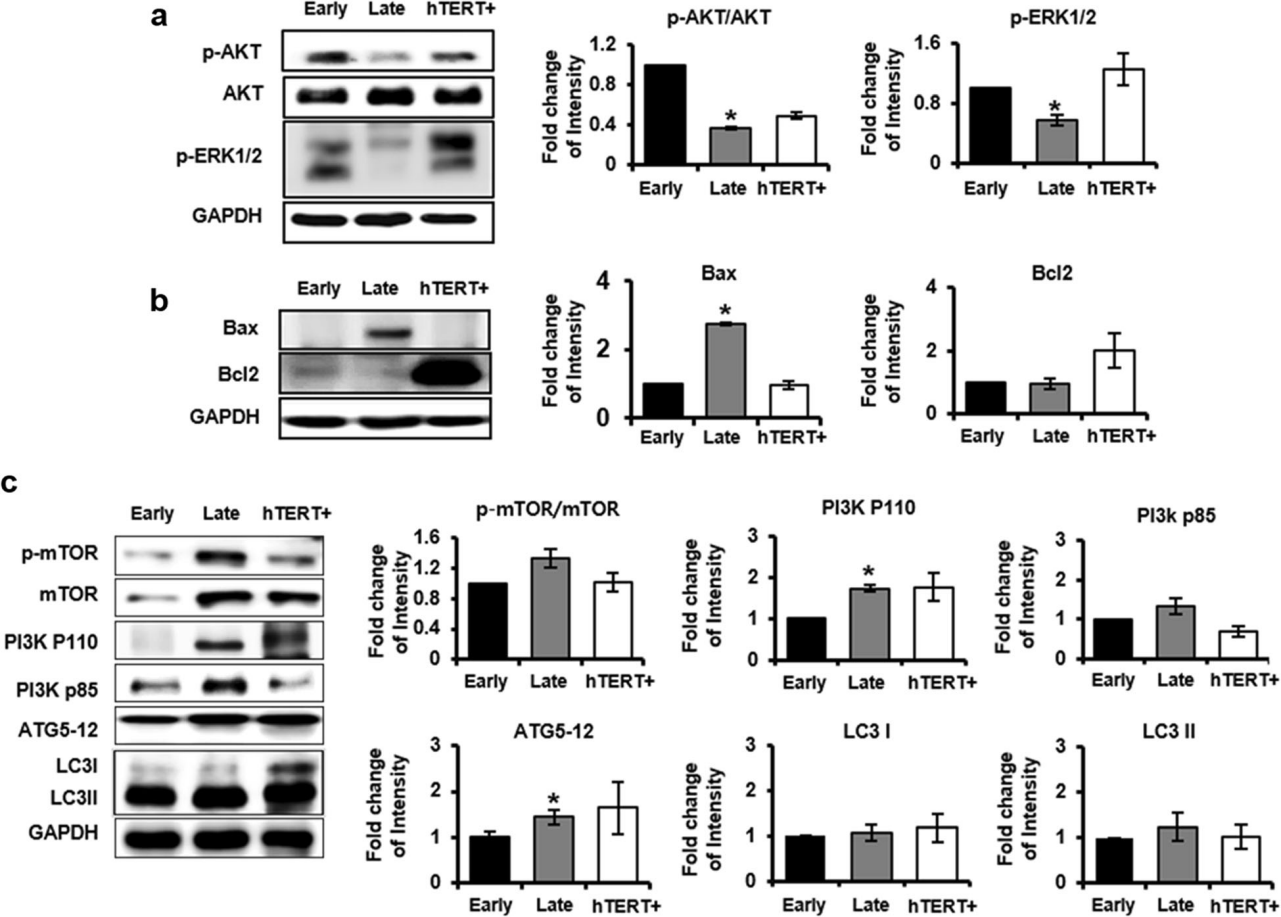
Effect of cell survival and death pathway in senescent PD-MSCs during long-term cultivation. **a** The expression of p-AKT/AKT and p-ERK1/2 gene related to survival pathway were analyzed by Western blot in early and late passage PD-MSCs. **b** The expression of Bax and Bcl2 gene related to pro-/anti-apoptosis regulator were analyzed by Western blot in early and late passage PD-MSCs. **c** The gene expression of p-mTOR/mTOR, PI3K-p100/-p85, ATG 5–12, and LC3 I/II related to autophagy pathway was analyzed by Western blot in early and late passage PD-MSCs. The data were representative of three independent experiments and presented by Image J software and expressed as means ± S.D. An asterisk indicates *P* < 0.05 versus early passage

Activation of Akt and ERK1/2 plays an important role in the regulation of cell survival and proliferation. To determine the effects of survival proteins during long-term cultivation of PD-MSCs, the expression and phosphorylation levels of Akt and ERK1/2 were determined using Western blot analysis. As shown Fig. 3a, phosphorylated-AKT(p-AKT)/AKT, a critical regulator of cell survival, represented a significantly decreased level in late passage compared to early and hTERT+ (*P* value = 0.006). In addition, the expression of p-ERK1/2 was significantly decreased in late passage compared to early and hTERT+ PD-MSCs (*P* value = 0.04). Therefore, gene expression related to proliferation and survival was significantly declined in late passage compared to early and hTERT+.

Next, we investigated the expression level of apoptosis and autophagy-related proteins in different passages of PD-MSCs using Western blot analysis. The expression of apoptotic molecule Bax was markedly increased (*P* value = 0.008), whereas antiapoptotic Bcl-2 expression was similar in both late and early passage PD-MSCs. Overexpression of Bcl-2 in hTERT+ PD-MSCs was observed to prevent cell death (Fig. 3b). Several autophagy-related proteins act in cells during aging. Mammalian target of rapamycin (mTOR) is a negative regulator of autophagy. As shown in Fig. 3c, the p-mTOR/mTOR level was slightly increased in late passage, and also downstream factors, PI3K and ATG5–12 (*P* value = 0.02), were increased, but slight increasing trend of LC3I/II level showed no significant statistical differences between early and late passages. These results suggest that senescent PD-MSCs according to long-term culture represent the possibility of affecting the initial process of autophagy. Therefore, further studies are required to clarify the mechanism of autophagy regulation in senescent PD-MSCs.

### Effect of mitochondrial dysfunction in senescent PD-MSCs during long-term cultivation

**Fig. 4:**
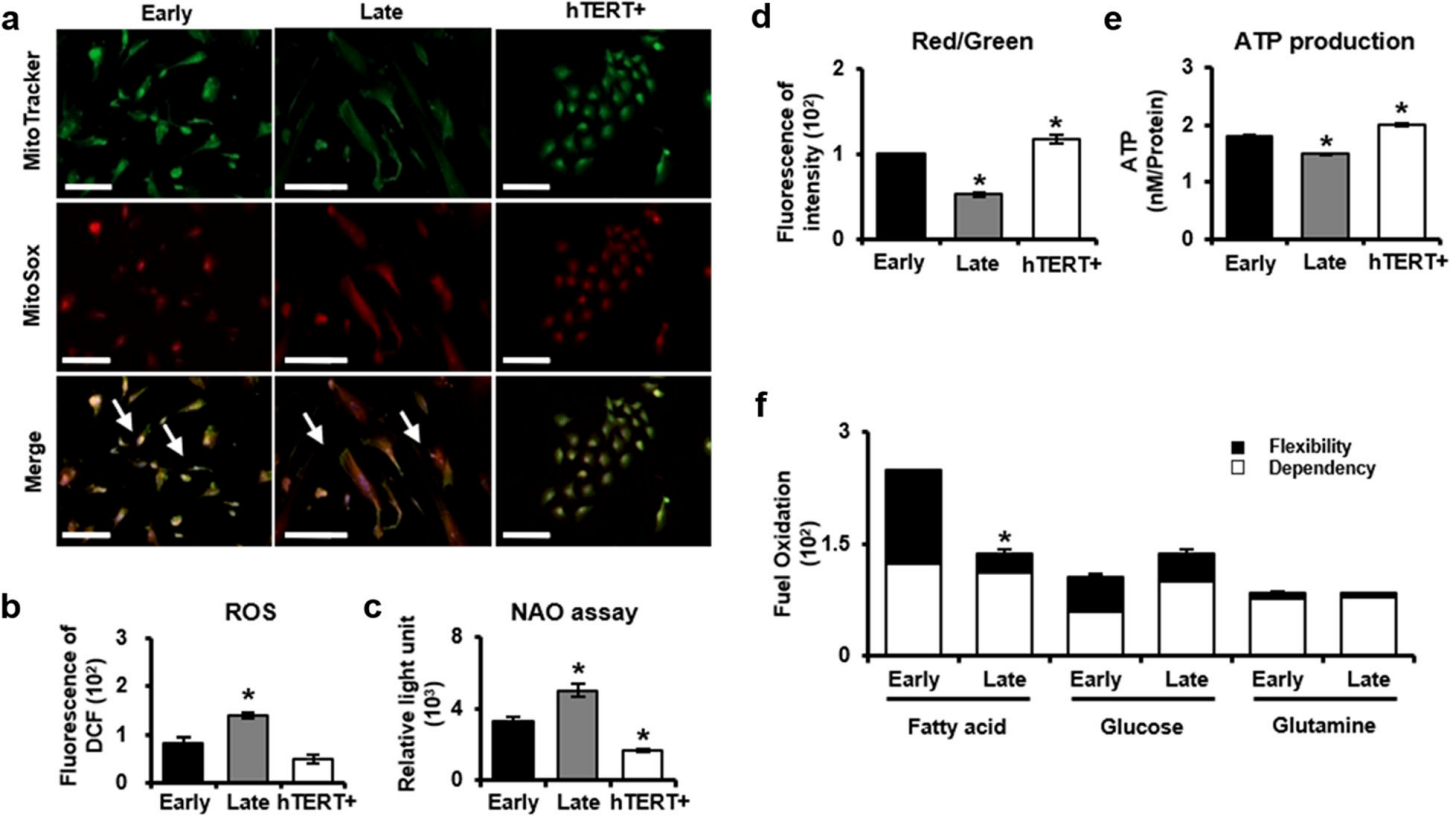
Effect of mitochondrial dysfunction in senescent PD-MSCs during long-term cultivation. **a** Representative confocal images showed mitochondrial superoxide levels (MitoSox Red) and mitochondrial content (MitoTracker Green) in early passage, late passage, and hTERT+ PD-MSCs (magnification, × 200). **b** The ROS levels of early and late passage PD-MSCs were measured with the fluorescent dyes DCFDA. **c** The mitochondrial mass of early and late passage PD-MSCs was analyzed by using the NAO, respectively. **d** The mitochondrial membrane potential of early and late passage PD-MSCs was detected by JC-1 fluorescent dye which is measured as the ratio of the J-aggregated (red) to the JC-1 monomeric (green) forms. **e** Relative ATP levels of early and late passage PD-MSCs were analyzed by ATP production assay kit. **f** XF analyses revealed the oxygen consumption rates of fatty acid, glucose, and glutamine pathways in early and late passage PD-MSCs. The data were representative of three independent experiments and expressed as means ± S.D. An asterisk indicates *P* < 0.05 versus early passage

Since it is well known that cellular senescence has been associated with alteration of mitochondrial function by oxidative stress such as reactive oxygen species (ROS) [10], we observed whether long-term cultivation would promote ROS production in PD-MSCs. The level and localization of ROS production in the mitochondria was determined by double staining with MitoTracker Green, which labels the mitochondria in a manner that is independent of the membrane potential, and MitoSox Red, which specifically stains mitochondrial superoxide (O_2_^−^) among ROS. As shown in Fig. 4a, it was confirmed that ROS was generated in the mitochondrial region by fluorescent overlap in these PD-MSCs. In late passage PD-MSCs, the ROS production as well as mitochondrial mass increased. To further clarify the ROS level, H_2_O_2_ accumulation was quantitatively examined by fluorescence microplate reader using DCFDA, which is a non-fluorescent DCFDA form a fluorescent DCF in the presence of ROS, especially H_2_O_2_. Consistently, late passage PD-MSCs showed about a 1.5-fold increase, whereas hTERT+ PD-MSCs showed a significant decrease in compared to early PD-MSCs (Fig. 4b). Next, we measured mitochondrial biogenesis in early and late passage PD-MSCs. To quantitatively clarify mitochondrial mass, we used NAO, which measures mitochondrial mass by binding to cardiolipin in all mitochondria. Similar to the morphology identified by MitoTracker, mitochondrial mass increased in late passage PD-MSCs compared with early cells (Fig. 4c). We then evaluated mitochondria metabolic functions by mitochondrial membrane potential (Δψm) and ATP production assays (Fig. 4d, e). Mitochondrial membrane potential was quantified by the JC-1 fluorescence dye. At low membrane potential, a cationic carbocyanine dye accumulates as a monomer in the mitochondria, which yield a green fluorescence (depolarization), while it aggregates at high membrane potential with a red fluorescence (hyperpolarization). Our results showed about 50% reduction (i.e., depolarization) in Δψm as a ratio of red/green fluorescence intensity in late passage PD-MSCs in comparison to early cells. However, hTERT overexpressed PD-MSCs showed higher hyperpolarization than early cells. Cellular ATP content was also decreased in late PD-MSCs similar to Δψm. Mitochondria produce ATP through electron transport chain (ETC) and oxidative phosphorylation (OXPHOS) in three major nutrients such as fatty acids, glucose, and amino acids. A “metabolic flexible” means free switch between major nutrients depending on nutritional and physiological cues [19]. A previous study demonstrated that senescent bone marrow MSCs evidenced metabolic inflexibility [20]. Accordingly, we investigated the changes in energy supply and metabolic flexibility during long-term cultivation in PD-MSCs by the XF Mito Fuel Flex system. The metabolic flexibility in media containing each major nutrient is low in replicative senescent PD-MSCs compared to early cells. Changes in the flexibility of fatty acids and glucose media in early PD-MSCs were more dynamic than those of glutamine, and dependency in these media was also consistent in late PD-MSCs, suggesting that periodic shifts in fatty acids and glucose are responsible for energy metabolism of PD-MSCs (Fig. 4f). Taken together, these results suggest that long-term cultivation causes mitochondrial dysfunction in mitochondrial membrane potential and metabolic flexibility following the morphological changes of mitochondria in PD-MSCs.

### Effect of increased CPT1A in senescent PD-MSCs during long-term cultivation

**Fig. 5:**
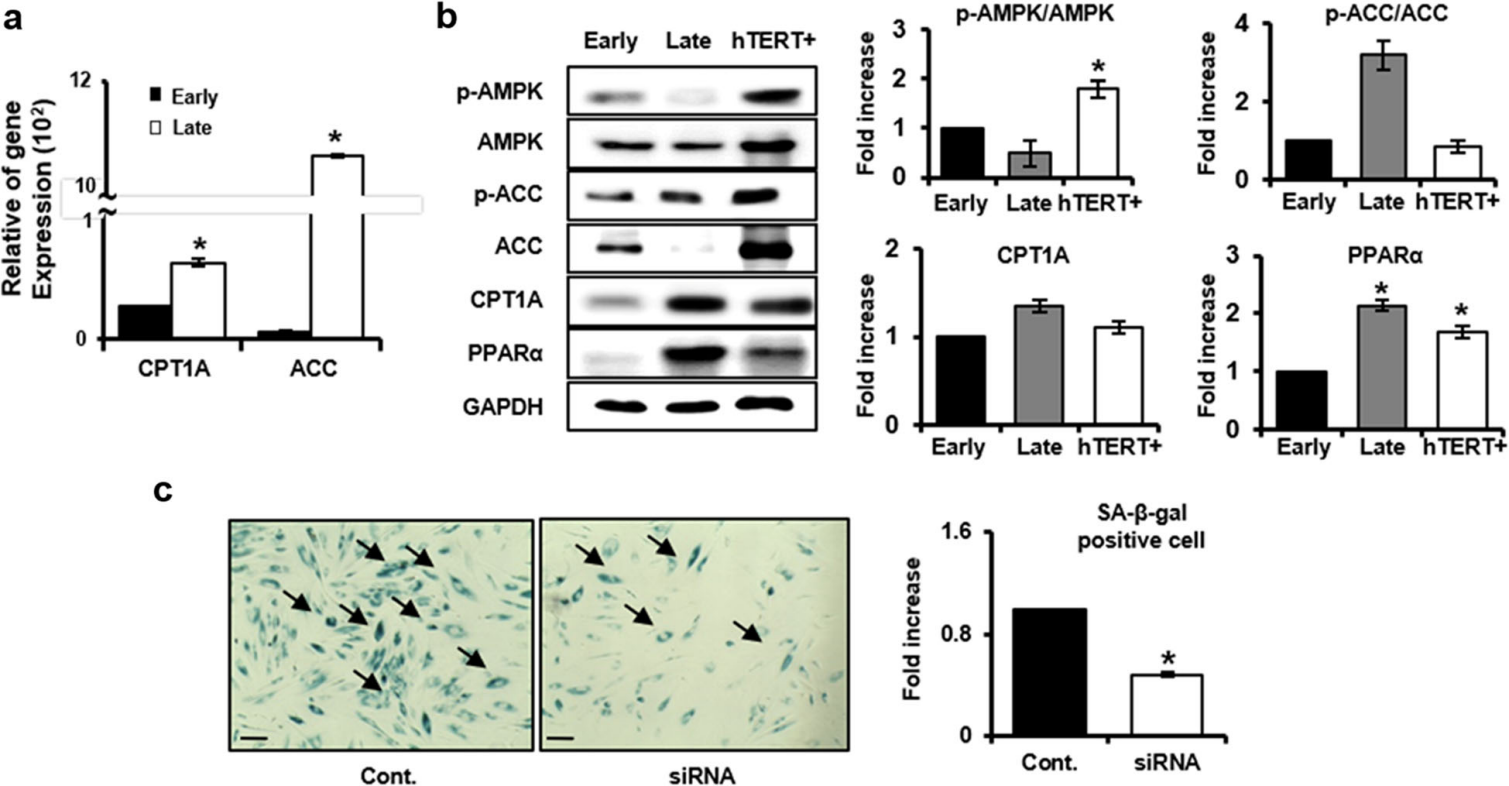
Effect of increased CPT1A in senescent PD-MSCs during long-term cultivation. **a** The levels of CPT1A and ACC mRNA were determined by using qRT-PCR. **b** Protein levels of p-AMPK/AMPK, p-ACC/ACC, PPARα, and CPT1A related to fatty acid pathway of early and late passage PD-MSCs were analyzed by Western blotting. **c** SA-β-galactosidase activity of late passage PD-MSCs treated with CPT1A siRNA was assayed by SA-β-galactosidase staining (scale bar, × 40). The SA-β-galactosidase stained cells were counted by Image J software. The data were representative of three independent experiments and expressed as means ± S.D. An asterisk indicates *P* < 0.05 versus early passage and control group

We investigated fatty acid oxidation (FAO)-related factors to confirm FAO pathway in PD-MSCs during long-term cultivation. In skeletal muscle cells, it is well established that AMP-activated protein kinase (AMPK) inhibits acetyl-CoA carboxylase (ACC) through phosphorylation, which reduces intracellular malonyl-CoA levels and stimulates carnitine palmitoyl transferase 1 (CPT1) and then increases the influx of long-chain fatty acids into the mitochondria where they are oxidized [21]. Quantitative RT-PCR revealed that late passage PD-MSCs increased dramatically the mRNA levels of ACC and CPT1A compared to early cells (Fig. 5a). In addition, Western blot analysis showed that the increase of CPT1A (*P* value = 0.06) was followed by the increase of expression of p-ACC versus total-ACC by long-term cultivation in PD-MSCs (*P* value = 0.053), while the phosphorylation level of AMPK, an upstream of ACC, was significantly decreased suggesting activation of AMPK is cell type-specific. Peroxisome proliferator-activated receptor (PPARα) is another major regulator of FAO as a transcription factor, which is predominantly expressed in tissues that oxidize fatty acids at a rapid rate, such as the liver, brown adipose tissue, heart, and kidney [22]. As expected, increased passages of PD-MSCs induced a significant increase of PPARα (*P* value = 0.02) (Fig. 5b). To further clarify the role of CPT1A increased by replicative senescent in PD-MSCs, we blocked CPT1A with the pharmacologic CPT1 inhibitor etomoxir or siRNA targeting CPT1A in late passage PD-MSCs and evaluated its effects on FAO signaling and senescence biomarker expression. We confirmed that the mRNA expressions of CPT1A and ACC were markedly inhibited, and the protein expression level of p-ACC as well as CPT1A was also reduced by treatment with etomoxir or siRNA in late passage PD-MSCs (Additional file 1: Figures S2a and S2b). Interestingly, inhibition of CPT1A reduced significantly SA-β-gal positive cells, indicating that fatty acid metabolism plays an important role in replicative senescence of PD-MSCs (Fig. 5c).

### Effect of downregulated CPT1A on mitochondrial activities in senescent PD-MSCs

**Fig. 6:**
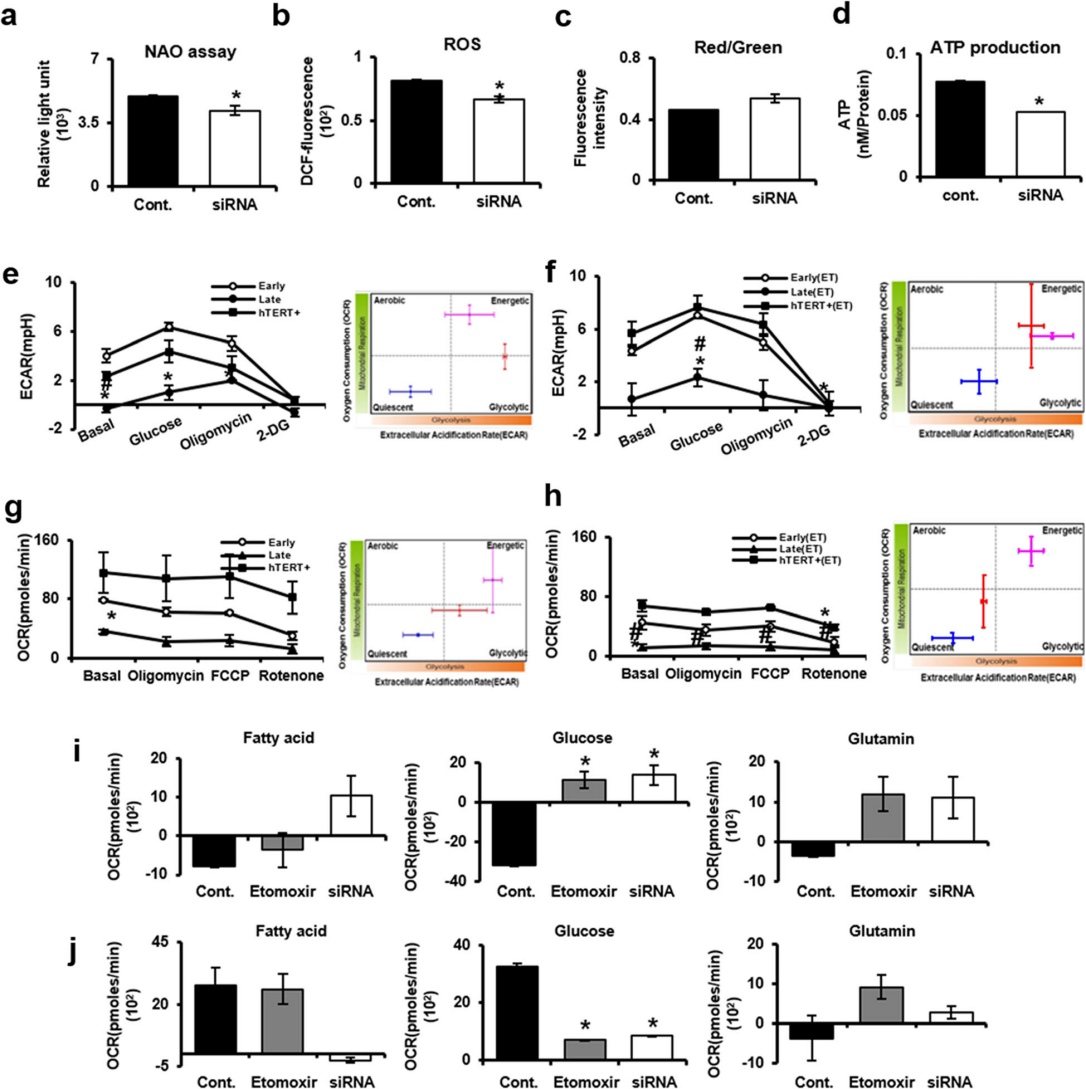
Effect of downregulated CPT1A on mitochondrial metabolism in senescent PD-MSCs. **a** To analyzed mitochondrial function of PD-MSCs with CPT1A siRNA, the mitochondrial mass, **b** the ROS levels, **c** mitochondrial membrane potential, and **d** ATP production were analyzed in late passage PD-MSCs treated with CPT1A siRNA such as CPT1A inhibitor compared to control. The glycolytic ability of senescent PD-MSCs treated with etomoxir such as CPT1A inhibitor was determined by XF24 analyzer. **e** The extracellular acidification rate (ECAR) of early and late passage PD-MSCs was analyzed by using glycolysis-XF assay kit and **f** also determined in late passage PD-MSCs treated with etomoxir. **g** The mitochondrial oxygen consumption rate (OCR) of early and late passage was determined by using mitochondrial stress-XF assay kit and **h** also determined after the treatment with etomoxir. **i** The oxygen consumption rates (OCR) of fatty acid, glucose, and glutamine pathways were analyzed in early passage PD-MSCs and **j** late passage PD-MSCs treated with siRNA and etomoxir. The data were representative of three independent experiments and expressed as means ± S.D. An asterisk indicates *P* < 0.05 versus early passage. Red, early; blue, late; pink, hTERT+. Red, control; blue, etomoxir; pink, CPT1A siRNA

To further specifically look at the importance of FAO metabolism in PD-MSCs, we subsequently investigated how the mitochondrial metabolic function changes by CPT1 inhibitor treatment in late passage PD-MSCs. Interestingly, treatment of CPT1A siRNA decreased ATP production despite the reduction of mitochondrial mass and ROS, including an increase of mitochondrial membrane potential, suggesting that fatty acid metabolism of PD-MSCs during long-term cultivation contributes more to ATP production than glycolysis (Fig. 6a–d). The glycolytic ability of senescent PD-MSCs was performed using XF24 Extracellular Flux analyzer. Oligomycin inhibits mitochondrial ATP and 2-DG inhibits glycolysis. So, XF-assay analyzed cells’ dependence on mitochondrial energy metabolism including glycolysis pathway. The glycolytic rate in late passage was significantly lower than those in early passage and hTERT+ PD-MSCs (Fig. 6e). We also confirmed decreased glycolytic activity in late passage after the treatment with etomoxir compared to early passage (Fig. 6f). Mitochondrial stress test was also performed to measure oxygen consumption rate (OCR) of senescent PD-MSCs. The results showed that late passage PD-MSCs have statistically significant lower OCR than early passage regardless of the treatment with etomoxir (Fig. 6g, h). These findings suggest that senescent PD-MSCs are associated with altered mitochondrial metabolism including fatty acid oxidation and glycolysis. Furthermore, the alterations in mitochondrial fuel usage, such as fatty acids, glucose, and glutamine, were determined by using XF Mito Fuel Flex system. Otherwise, in late passage PD-MSCs (Fig. 6j), the OCRs in fatty acid and glucose pathways except glutamine pathway were higher than those of early passage (Fig. 6i). Interestingly, etomoxir treatment on fatty acid pathway in late passage markedly increased the OCR level, whereas the knockdown of CPT1A with siRNA on fatty acid pathway in late passage significantly decreased the OCR level compared to early passage. These results suggest that fatty acid is the most influential factor among alterations in mitochondrial fuel usage, and the expression of CPT1A plays an important role in mitochondrial function. In addition, CPT1A can lower the OCR which is the mitochondrial stress level in senescent PD-MSCs.

## Discussion

The limited self-renewal of MSCs is one of obstacles to overcome in the development of stem cell-based therapy in degenerative medicine, although they have multi-lineage differentiation potential and immunomodulatory effect [23]. Cellular stresses can trigger shortness of hTERT in cells and induce replicative senescence. Accordingly, to investigate cellular senescence and related mechanism during long-term cultivation in PD-MSCs, we used PD-MSCs with different passages and hTERT+ PD-MSCs established in our previous study [24] as a positive control. In our present study, we demonstrated that PD-MSCs during long-term cultivation undergo senescence, which is characterized by a lowered differentiation potential and a reduced proliferation rate by G1 cell cycle arrest via p21 in a p53 independent pathway with the same result as previous research [25], although it differs from some studies that the p53/p21 pathway plays a role in cell growth arrest, cellular senescence, and apoptosis [26, 27]*.* Several studies reported that overexpression of hTERT helps PD-MSCs escape from replicative senescence suggesting that hTERT plays an important role in replicative senescence. Interestingly, the differentiation potential of hTERT+ has a difference for adipogenic and osteogenic compared to other cells. In previous reports, Ikebale et al. reported that immortalized stem cells by TERT gene transfection shows lower potential for adipogenic differentiation [28, 29]. These data we matched with our results as Fig. 2b in the study.

Mitochondria are highly dynamic organelles, which can regulate through the process of fission and fusion, allowing them to adjust their size and shape during apoptosis, autophagy, mitochondrial biogenesis, adipogenic differentiation of human mesenchymal stem cells (hMSCs), and cellular senescence [10, 30]. Senescence can be caused by cellular stresses, especially, ROS-mediated oxidative stress induces replicative senescence leading to lipid peroxidation, mitochondrial dysfunction, energy failure, and metabolic disturbance in the cell membrane [31, 32]. Mitochondria are also energy factories to maintain and regulate various cellular processes and their functions are critical to cell survival [33]. In the mitochondrial fuel selection, glucose has been generally regarded as the primary energy source rather than lipids and amino acids, as glucose supply is constant, consistent, and reliable. However, fatty acid metabolism has been found to play an important role during human pregnancy and in placental growth by the activity and expression confirmation of FAO enzymes in the human placenta [[17]. Recently, it has been also reported that fatty acid metabolism is associated with the aging of organelles with aging and the senescence of bone marrow MSCs [20, 34]. Similarly, our study demonstrated that metabolic activity of fatty acids was higher than that of glucose or glutamine in both early and late passages, but late passage PD-MSCs exhibited inflexible compared with early passage cells. To specify more details, we investigated signaling pathway underlying FAO. As expected, the PPARα, p-ACC, and CPT1A associated with fatty acid metabolism increased in late passage. Interestingly, the CPT1A knockdown reversed mitochondrial dysfunction (decreased ROS, NAO, and improved mitochondrial membrane potential) and inhibited senescence induced by long-term cultivation in PD-MSCs, indicating that increased CPT1A in senescent PD-MSCs triggers mitochondrial dysfunction by unbalanced energy metabolism as well as anti-aging effect by downregulated CPT1A gene and that FAO has a significant impact on the senescence of PD-MSCs. This is similar to the previous reports that the specific increase in saturated fatty acids is a characteristic of many age-related human diseases including cardiovascular disease and cancer, and that saturated fatty acid metabolism is key link between cell division, cancer, and senescence in cellular and whole organism aging [34, 35]. However, ATP production did not restore as much as early PD-MSCs despite the increased mitochondrial membrane potential. It is considered that the replicative senescent PD-MSCs have relatively metabolic inflexibility compared to early passage cells. In addition, the inhibition of FAO with etomoxir or CPT1A siRNA in replicative senescent PD-MSCs showed no change in glutamine metabolism and glycolysis shifting for energy production, which means that they enable exquisite crosstalk and cooperation between fatty acid and glucose to maintain energy as in Randle’s glucose-fatty acid cycle hypothesis (Additional file 1: Figure S3a, b, c, d and e) [36]. Some studies have indicated an increase in glucose uptake during oncogene-induced senescence (OIS), and cancer cells preferentially use glycolysis under aerobic conditions [37, 38]. Accordingly, it may also be thought that higher aerobic glycolysis than anaerobic during long-term cultivation promotes senescence of PD-MSCs, although it is cell type-specific. Further studies on metabolic alterations are needed to clearly understand the senescent process of PD-MSCs. Taken together, our findings first showed that CPT1A plays an important factor in mitochondria function via regulation of energy metabolism, and ROS level in replicative senescence of PD-MSCs according to long-term cultivation. These data help us understand the fundamental mechanism of self-renewal of PD-MSCs and support to overcome the replicative senescence of PD-MSCs.

## Conclusions

In this study, PD-MSCs during prolonged in vitro culture undergo senescence exhibiting reduced proliferation rate by G1 arrest and lowered differentiation potential, and overexpression of hTERT helps PD-MSCs escape from replicative senescence. Also, senescent PD-MSCs according to long-term culture represent the possibility of affecting the initial process of autophagy. Senescent PD-MSCs appeared mitochondrial dysfunction in mitochondrial membrane potential and metabolic flexibility following the morphological changes of mitochondria. In addition, CPT1A decreased the OCR which is the mitochondrial stress level in senescent PD-MSCs. Therefore, our findings showed that CPT1A plays a role as an essential factor in mitochondria function via control of energy metabolism, and ROS level in replicative senescence of PD-MSCs through long-term cultivation.

## Supplementary information

**Additional file 1: Figure S1.** S1Characterization related to tumor suppressor gene expression in PD-MSCs during long-term cultivation. The p53 gene expression related to tumor suppressor in Early and Late passage PD-MSCs was assayed by western blotting. The data were representative of three independent experiments and expressed as means ± S.D. * indicates *P*<0.05 versus Early passage. **Figure S2.** Effect of fatty acids in senescent PD-MSCs according to CPT1A inhibition. **a** The levels of CPT1A and ACC mRNA were analyzed in Late passage PD-MSCs with Etomoxir and siRNA-CPT1A treated group by using qRT-PCR. **b** The protein levels of p-ACC/ACC ratio and CPT1A were assayed in Late passage PD-MSCs treated with Etomoxir by using western blotting. The data were representative of three independent experiments and expressed as means ± S.D. * indicates *P*<0.05 versus Non-treated Late passage PD-MSCs. **Figure S3.** Effect of mitochondrial metabolism in PD-MSCs according to CPT1A inhibition through Etomoxir treatment. **a** The Extracellular acidification rate (ECAR) of Early and Late passage PD-MSCs were analyzed by using glycolysis-XF assay. **b** Mitochondrial oxygen consumption (OCR) of Early and Late passage PD-MSCs were analyzed by using mitochondrial stress-XF assay. **c** The mitochondrial fuel levels of senescent PD-MSCs with siRNA CPT1A were analyzed according to inhibition of fatty acid, **d** glucose and **e** glutamic pathway by using XF24 analyzer. The data were representative of three independent experiments and expressed as means ± S.D. * indicates *P*<0.05 versus Non-treated group. ** indicates p>0.05 versus in group (e.g., control and siRNA).

## Data Availability

The data that support the findings of this study are available from the corresponding author upon reasonable request.

## Abbreviations

ACC: Acetyl-CoA carboxylase
CPT1: Carnitine palmitoyl transferase 1
ETC: Electron transport chain
FAO: Fatty acid oxidation
hMSCs: Human mesenchymal stem cells
OCR: Oxygen consumption rate
OIS: Oncogene-induced senescence
OXPHOS: Oxidative phosphorylation
PD-MSCs: Human placenta-derived mesenchymal stem cells
PPARα: Peroxisome proliferator-activated receptor
ROS: Reactive oxygen species
SA-β-Gal: Senescence-associated β-galactosidase
