# Supplementary figures and images for: Alteration of fatty acid oxidation by increased CPT1A on replicative senescence of placenta-derived mesenchymal stem cells
Source: Stem Cell Res Ther. 2020 Jan 3;11:1. doi: 10.1186/s13287-019-1471-y (PMC6941254; doi:10.1186/s13287-019-1471-y)

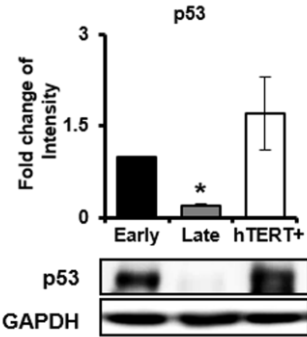

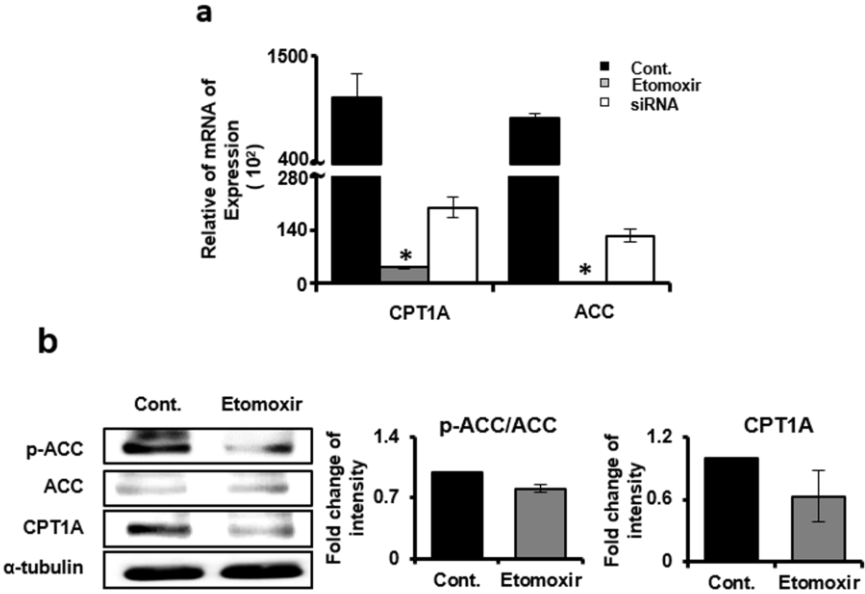

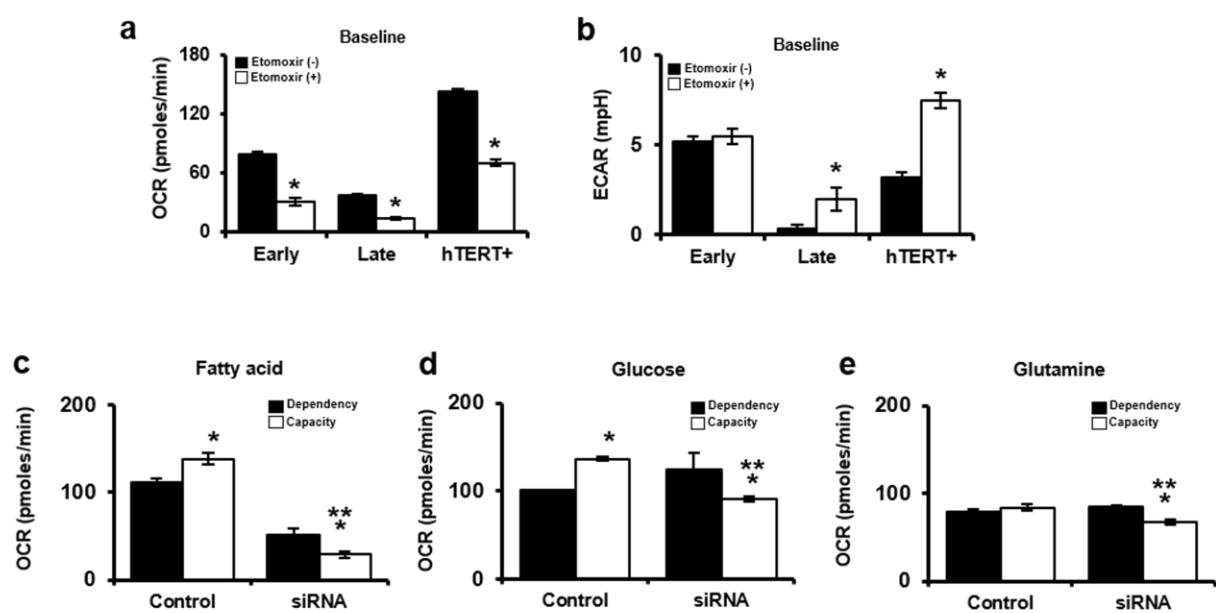

Supplement: Supplementary file 1 — Additional file 1: Figure S1. S1Characterization related to tumor suppressor gene expression in PD-MSCs during long-term cultivation. The p53 gene expression related to tumor suppressor in Early and Late passage PD-MSCs was assayed by western blotting. The data were representative of three independent experiments and expressed as means ± S.D. * indicates P<0.05 versus Early passage. Figure S2. Effect of fatty acids in senescent PD-MSCs according to CPT1A inhibition. a The levels of CPT1A and ACC mRNA were analyzed in Late passage PD-MSCs with Etomoxir and siRNA-CPT1A treated group by using qRT-PCR. b The protein levels of p-ACC/ACC ratio and CPT1A were assayed in Late passage PD-MSCs treated with Etomoxir by using western blotting. The data were representative of three independent experiments and expressed as means ± S.D. * indicates P<0.05 versus Non-treated Late passage PD-MSCs. Figure S3. Effect of mitochondrial metabolism in PD-MSCs according to CPT1A inhibition through Etomoxir treatment. a The Extracellular acidification rate (ECAR) of Early and Late passage PD-MSCs were analyzed by using glycolysis-XF assay. b Mitochondrial oxygen consumption (OCR) of Early and Late passage PD-MSCs were analyzed by using mitochondrial stress-XF assay. c The mitochondrial fuel levels of senescent PD-MSCs with siRNA CPT1A were analyzed according to inhibition of fatty acid, d glucose and e glutamic pathway by using XF24 analyzer. The data were representative of three independent experiments and expressed as means ± S.D. * indicates P<0.05 versus Non-treated group. ** indicates p>0.05 versus in group (e.g., control and siRNA). [file 13287_2019_1471_MOESM1_ESM.pdf]
